# Smart Distribution Boards (Smart DB), Non-Intrusive Load Monitoring (NILM) for Load Device Appliance Signature Identification and Smart Sockets for Grid Demand Management

**DOI:** 10.3390/s20102900

**Published:** 2020-05-20

**Authors:** See Gim Kerk, Naveed UL Hassan, Chau Yuen

**Affiliations:** 1 Engineering Product Development Pillar, Singapore University of Technology and Design, Singapore 487372, Singapore; seegim_kerk@mymail.sutd.edu.sg; 2 Department of Electrical Engineering, Syed Babar Ali School of Science and Engineering, Lahore University of Management Sciences (LUMS), Sector U, DHA, Lahore 54792, Pakistan; naveed.hassan@lums.edu.pk

**Keywords:** Home-ESS, smart distribution board, smart sockets, grid stability

## Abstract

Traditionally, the choices to balance the grid and meet its peaking power needs are by installing more spinning reserves or perform load shedding when it becomes too much. This problem becomes worse as more intermittent renewable energy resources are installed, forming a substantial amount of total capacity. Advancements in Energy Storage System (ESS) provides the utility new ways to balance the grid and to meet its peak demand by storing un-used off peak energy for peak usage. Large sized ESS—mega watt (MW) level—are installed by different utilities at their substations to provide the high speed grid stabilization to balance the grid to avoid installing more capacity or triggering any current load shedding schemes. However, such large sized ESS systems and their required inverters are costly to install, require much space and their efficacy could also be limited due to network fault current limits and impedances. In this paper, we propose a novel approach and trial for 3000+ homes in Singapore of achieving a large capacity of demand management by developing a smart distribution board (DB) in each home with the high speed metering sensors (>6 kHz sampling rate) and non-intrusive load monitoring (NILM) algorithm, that can assist home users to perform the load/appliance profile identification with daily usage patterns and allow targeted load interruption using the smart sockets/plugs provided. By allowing load shedding at device or appliance level, while knowing their usage profile and preferences, this can allow such an approach to become part of a new voluntary interruptible load management system (ILMS) that requires little user intervention, while minimizing disruption to them, allowing ease of mass participation and thus achieving the intended MW demand management capacities for the grid. This allows for a more cost effective way to better balance the grid without the need for generation capacity growth, large ESS investment while improving the way to perform load shedding without disruptions to entire districts. Simply, home users can now know and participate with the grid in interruptible load (IL) schemes to target specific home appliance, such as water heaters or air conditioning, allowing interruptions during certain times of the day, instead of the entire house, albeit with the right incentives. This allows utilities to achieve MW capacity load shedding with millions of appliances with their preferences, and most importantly, with minimal disruptions to their consumers quality of life. In our paper, we will also consider coupling a small sized Home Energy Storage System (HESS) to amplify the demand management capacity. The proposed approach does not require any infrastructure or wiring changes and is highly scalable. Simulation results demonstrate the effectiveness of the NILM algorithm and achieving high capacity grid demand management. This approach of taking user preferences for appliance level load shedding was developed from the results of a survey of 500 households that indicates >95% participation if they were able to control their choices, possibly allowing this design to be the most successful demand management program than any large ESS solution for the utility. The proposed system has the ability to operate in centralized as part of a larger Energy Management System (EMS) Supervisory Control And Data Acquisition (SCADA) that decide what to dispatch as well as in autonomous modes making it simpler to manage than any MW level large ESS setup. With the availability of high-speed sampling at the DB level, it can rely on EMS SCADA dispatch or when disconnected, rely on the decaying of the grid frequency measured at the metering point in the Smart DB. Our simulation results demonstrate the effectiveness of our proposed approach for fast grid balancing.

## 1. Introduction

In a drive towards enhancing grid stability/optimization and to meet growing energy demand, many approaches are being adopted from distributed generation, advanced SCADA controls, load shedding and most recently, demand management and Energy Storage Systems (ESS) [1,2,3,4,5,6,7,8,9,10,11,12,13]. For grid stability, power generation and load demand have to be matched at all the times. As the peak load demand on the grid is increased, traditional utilities either increase the overall generation capacity or perform high speed under-frequency load shedding using substation relays [5,6,7,8,9]. Both these traditional methods have their own disadvantages. Increasing the overall generation capacity to meet peak load demand which only lasts up to few hours in today’s context, is highly expensive, wasteful, and environmentally unsustainable [8,9]. On the other hand, load shedding at the substation level can be hugely disruptive and inconvenient as it affects thousands of homes and businesses and may also adversely affect the economy if it becomes too frequent [6,7,8,9]. Load shedding is not a voluntary choice and usually adopted as a last resort to avoid a cascading fault and will attract huge penalties by the energy regulator. Set to shed population centers first, followed by commercial, industrial, financial institution loads and finally critical loads such as hospitals, military and government facilities, each outage event will incur millions in penalties, huge losses to the economy which it serves and that does not include the damages to the end customers, if they are proven negligent. Last but not least, the reliability and quality of electricity supply can lead to hundreds of millions of losses in the economy and possibly lives. Hence, any methods that avoid mass load shedding and in enhance the quality of supply is always preferred.

In recent years, there has been a great interest in renewable energy generation to meet the growing load demands in an environmentally friendly manner [7,8]. The growth in renewables, while encouraging, further exacerbate grid stability issues and increase system faults, with many pushing for more spinning reserve capacities to negate any volatility, which negates the benefits of this type sustainable energy source. With the advent of low cost communication technologies, high speed metering with more powerful edge computing capacity and Internet-of-Things (IOT) connectivity offered by telecoms, demand response management also becomes a tool to manage supply-demand balance [7,8,9]. In demand response management, with the help of different pricing signals or frequency change, loads can be tripped off instantly to meet the generation capacity via voluntary load interruptions. However, without large scale user participations, demand response management in this approach has a limited impact in resolving the grid stability issues [10], utilities have to resort to the unfavorable and traditional method of under-frequency load tripping when it fails to balance at the substation level to maintain stability, affecting tens of thousands of households with outages [10,11].

The emergence of utility level ESS provides another alternative approach to address the same concerns on peak demand growth, renewable intermittency and system balancing during generation or capacity loss [9,10,11,12,13]. In this approach, large sized ESS in MW are installed at the substation levels. A typical MW level ESS installation at a substation, that can be at the distribution or transmission level (66 kV and above), consists of the battery, which can be lithium ion, flow batteries and any other High Power or High Energy types. High Power types are categorized by ability to supply high power in MW but for very short durations, typically from a few seconds to less than 30 min for frequency stabilization and High Energy types for less than MW levels and for duration longer than 4 h for demand management. 

**Figure 1:**
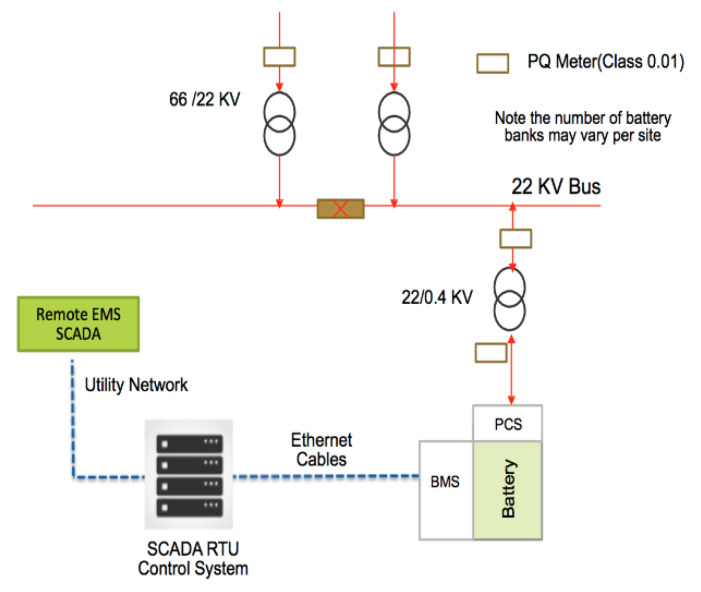
Typical Setup of a substation level Energy Storage System (ESS).

Next, it is the Power Conversion System (PCS) inverts the DC power to AC and finally, the Battery Management System (BMS) that manages the battery to avoid over-discharge or any thermal runaway. A SCADA EMS system is usually linked to manage the grid and perform load dispatches for the discharging and charging of the battery, considerations are usually on the network loading for discharging and for charging, in an open electricity market such as Singapore, spot electricity prices. A typical load at the 22 KV station in Singapore can vary between 7–12 MVA, about half of a typical 25 MVA transformer. To make any impact, the ESS must be at least 20–30% of the rated load or 1.4 to 3.6 MW power and a backup duration for at least 2 h. Depending on the technology, the space requirements can vary substantially and cost of setup can be substantial assuming there is availability of space for installation, a premium in all urban cities such as Singapore. While it is not uncommon to see many on-going trials with MW batteries [13,14], the widespread adoption of this solution cannot be expected unless the cost reduces to a level that can justify the investments in capital, land and even re-laying the cabling infrastructure to manage the higher current system. A typical installation design is as shown below at Figure 1, where the battery with its attached PCS are connected to the 22 KV bus and the Supervisory Control And Data Acquisition (SCADA) Remote Terminal Unit (RTU), which links it to the Remote Energy Management System (EMS).

In this paper, leveraging the advancements and cost reductions in high speed CT metering, the availability of low cost home gateways with high speed computing power, smart sockets with built-in breakers and low cost communication technologies, we propose a utility managed Home Energy Management System (HEMS) that provides appliance level smart load identification, curtailment and together with a small Home Energy Storage System (HESS), to assist in managing grid supply and demand mismatch. This approach relies on smart load identification at the house level based on a Non-Intrusive Load Monitoring (NILM) technique, which is a load signature identification application that resides in the smart gateway located in the new generation smart distribution box in the house. It automatically identifies the appliance when it is turned on and where it was turned on at each smart socket devices. This allows the system to provide users with the information of the usage profile with respect to Time of Use (TOU) and cost attributed to each house appliance and makes it possible for the system to control the load. 

At the same time, if we combine the HESS, we can discharge it to remove some of the loadings from the grid without impacting the grid, rotating across the households to maintain the “curtailed” capacity. The options of demand management, managing user preferences, reducing user inconveniences, while providing power for critical power to critical applications such as elderly and home patient care, creates the foundation for this approach to enhance grid stability via home demand management. 

**Figure 2:**
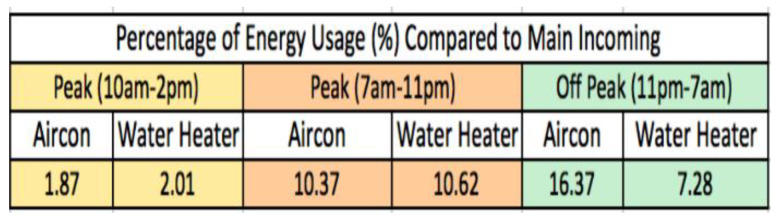
Survey summary results of 500 households.

A recent survey, prior to this actual project, was conducted for 500 homes for 2 to 3 bedrooms public housing flats (between 900–1200 square feet) to measure demand management and IL opportunities in performing appliance level load shedding. This type of public flat represents 80–90% of all housing types in Singapore. The measurements were done by only using an incoming single phase Time of Use (TOU) clamp on meter and smart plugs with built in metering to measure the total and selected aircon and heater loads for at least 7 days each. The following Figure 2 shows the breakdown in usage of the aircon and heater loads during the peak and off-peak periods.

The results show that an average of around 44.65% of total usage of each household is for aircon and heater loads. It is also found that around 80% of the households in the study group have air conditioning loads and 100% of them have water heaters, forming a substantial capacity that offers possible demand management capacity. A sub-breakdown for peak between 10 am to 2 pm was shown to show the capacity of usage that can offer the most useful interval for peak demand management.

A post survey shows that substantial percentage of 70.05% is willing to experience some inconvenience for up to 2 h of interruption a day (of no less than 30 min per interruption) if an acceptable discount is given off their overall daily usage. The figure increases to 95% if we allow them to choose what and when the load will be interrupted even for the same discount. From this survey, we can easily see that we will garner a higher demand management capacity if we have a solution that is intelligent enough to further identify smaller loads, other than the larger heater and aircon loads, to allow home user preferences to be further considered for minimizing their inconveniences. This forms the basis of developing the concept of smart DB to identify more load appliances (>100 W) for demand management purposes. With this design and proof of willingness and availability of excess capacities in each home, it allows the possibility of trading excess and voluntary load shedding in a secondary market where industrial and commercial buildings, could possibly buy additional demand capacities for a short period of time by offering an incentives for home consumers to inhibit or interrupt, without too many inconveniences to their overall quality of supply. Currently, all building contract capacities can only be changed annually, a very rigid and ineffective process that does not allow for a more efficient way to respond to actual production need. This will allow home consumers, with interruptible loads, who are willing to offset that increase by agreeing to turn off their loads based on a price that can accept, thus giving building owners a price to pay for taking it, and this is akin to sharing some of the penalties in certain fractions, which would otherwise be fully incurred to the utility. This will lead to a more efficient marketplace, an incentive program that encourage demand management operators to provide the investments of ESS in each home with no space constraints, high setup costs and when combine with IL, provide a much higher capacity per house for incentives to be shared between building owners, home users and the operators. This gives the utility more choices to provide different way to balance the grid, avoid catastrophic load shedding at the substations, allowing for a more stable grid, without much grid investment, that leads to lower costs and higher reliability to all stakeholders. 

Our contributions in this paper includes:

- Developing a smart distribution board (DB) with high speed CT metering sensor at >100 samples/cycle, NILM algorithm inside the smart DB home gateway can sense and disaggregate each appliance and smart sockets (SSO) with breaker controls that can turn off the load.
- Proposing a simple methodology of home load shedding system by aggregating a large number of identifiable and interruptible individual appliance load across households for utility demand management.
- Developing an HEMS that allows for easy home participation, takes in users preferences for IL while being part of a larger load shedding system, that can receive direct dispatch for grid stabilization. potentially can lead to higher IL participation.
- Integrating an HESS to the smart DB, to show that by doing so can substantially increase the distributed ESS capacity throughout the utility, lowering overall cost per MW installed as compared to utility substation ESS, while working in tandem with the HEMS to minimize load interruptions, user inconvenience and providing Uninterruptible Power Supply (UPS) supply for critical applications and services.
- Designing this utility managed HEMS as part of an SCADA EMS and ILMS that can also operate autonomously and will not require real time updates and dispatch simply by sensing the grid frequency at its point of coupling to decide the amount of load shedding or energy discharge from the HESS.

The rest of the paper is organized as follows. In Section 2 we discuss our proposed Smart DB system model, Home-ESS modeling is discussed in Section 3, HEMS including the design requirements of smart DB, NILM and smart sockets are discussed in Section 4, centralized and autonomous modes for HEMS aggregation employed for grid stabilization are described in Section 5, a case study is presented in Section 6, while the paper is concluded in Section 7.

## 2. Smart DB System Model 

In this system, we have designed the smart DB system based on a distributed nature, which will make individual connections to the main SCADA EMS using a device management platform. Connectivity is provided via the resident home router, which will provide the Internet connection. The Smart DB will utilize CT sensors to perform the high-speed data capturing or metering to all the outgoing circuits and perform the NILM algorithm to identify the load/appliance. Using broadband powerline (BPL) or any other available communications as the sensor network, the Smart DB will then communicate with the smart sockets with built in breakers that can be used to curtailed the selected load.

Each HEMS will be connected to the EMS SCADA cloud and users can understand their individual appliance usage and specify their load shed preferences using a smart phone application. The Smart DB, with the high speed CT, and a Raspi 3, acting as a smart home gateway, will measure the following parameters:

- system frequency
- active and reactive power
- Total and individual appliance energy usage including the aircon, heater and other loads (>150 W)

The HEMS, installed inside the Raspi 3 gateway, comes with a quad core CPU and on-board 16GB storage that runs the NILM algorithm for load identification, manage the curtailment of all loads based on user’s preferences. Users interact with the HEMS and Cloud system via their smart phone apps.

For grid stabilization, HEMS can operate in two modes (i) centralized mode and (ii) autonomous mode. In centralized operation, HEMS accepts and executes dispatch commands from the EMS SCADA and when it is not available, in autonomous mode, based on previously configured limits. With these limits, the local algorithm makes decisions, to dispatch Home-ESS or trip the appliance loads, based on the rise or decay of the high-speed sampling of the system frequency measured at the Smart DB to balance the grid much like an under-frequency relay. To ensure uniformity across all the HEMS, the EMS SCADA provides frequency thresholds for Home-ESS self-discharge and load curtailment to each load priorities as set by the users. This approach greatly enhances the stability of the approach by allowing it to operate with or without constant connection to the EMS SCADA.

**Figure 3:**
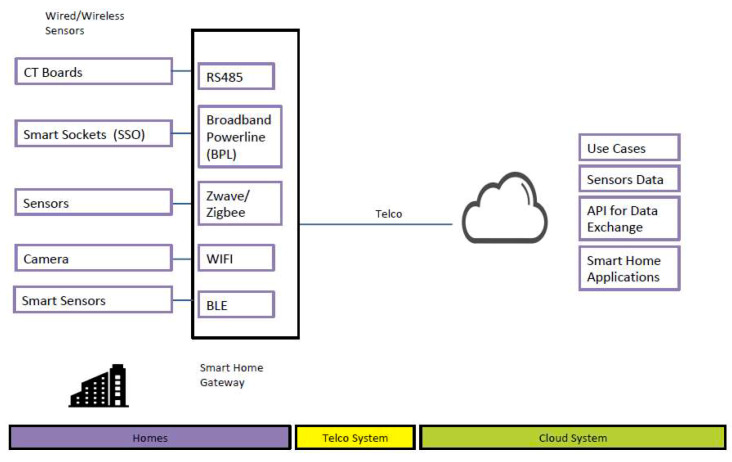
Connectivity of sensors within the house.

The Smart DB, are equipped with different communications options other than BPL to include Zwave, BLE and WIFI to allow it to integrate with other smart sensors. The connectivity from the gateway to all the sensors are shown in Figure 3 below.

As mentioned, the proposed system design of HEMS with HESS requires little or no modifications of the home wirings unlike large substation MW ESS installations, i.e., a million homes with 10 kWh storage with an average of 2 kW of IL at any time is 10,000 MWh with 2000 MW of loads that can tripped for frequency stabilization and a 10,000 MWh high power ESS for demand management, which is very expensive to implement, with no modifications to the grid. 

## 3. Home-ESS Modeling 

Many types of batteries have touted long lifespan and cycle life, but they either suffer from low round trip efficiency (flow batteries) or have high thermal runaway risks (lithium) [15]. At the grid transmission and distribution system levels, ESS options are very limited. Large batteries tend to be more expensive, require much space, require MW level inverters, and also have high maintenance costs [16,17,18,19]. In short, other than battery costs, non-battery cost considerations for large ESS are

- ESS equipment
- Space and network infrastructure
- Engineering and labor
- System setup, management and maintenance
- Part Replacements

On the other hand, Home-ESS batteries are smaller in size, are relatively low costs, with low power (<5 kW) inverters that are readily available in the market. However, several different types of battery technologies (lead-acid, lithium ion or saltwater battery) exist and they have their own advantages/disadvantages when used as Home-ESS.

A direct cost comparison study for our HESS approach against a typical setup at 22 KV system must first consider that High Power ESS systems have lower costs per kW and higher costs per KWh while High Energy Types have lower costs per kWh but higher costs per kW. Hence, the cost of a battery system can be calculated by looking at the power capacity or energy capacity costs, depending on the intended application. Consider the reference pricings given by U.S. Energy Information Administration (EIA) EIA 860, Annual Electric Generator Report for 2013–2016. Prices may have differed by now but it is still a good reference. 

Consider a typical High Power ESS 10 MW 4MWh setup (which is considered short duration since it is for <30 min), the power capacity cost is around USD1000/kW while using the energy capacity cost for short duration will be relatively higher at USD 2000–3400/kWh. Conversely, a 4 MW, 10 MWh high Energy ESS would have cost between USD 400–800/kWh for the energy capacity cost between USD1500–4200/kW. The estimated costs based on the weighted average cost would be around >10 MUSD for the High Power while the High Energy type will cost >8 MUSD just for the ESS equipment. The installation and related systems cost can be easily set at USD 3–4 MUSD for either solution. Land and transmission network cable upgrade can be so prohibitive that it will cost several times the cost for large ESS setup, even if the space is available. Except for 10+ sites out of 11,000+ substations, rollout of such solutions is not possible due to land constraints in Singapore. This situation is probably similar in any high population load centers where capacity is most needed. 

**Figure 4:**
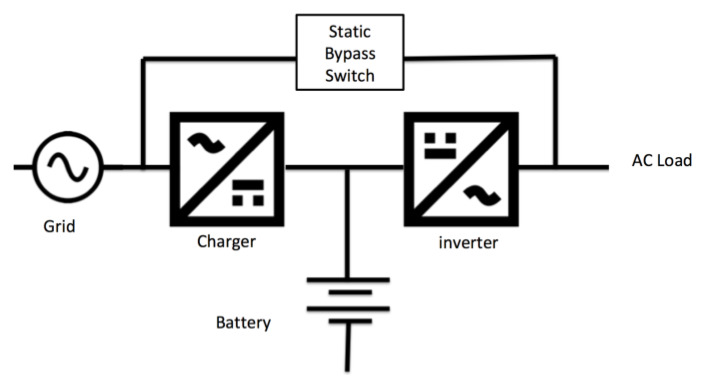
Typical Uninterrupted Power Supply (UPS) setup.

Consider the HESS alternative where the average power requirement of a typical household of around 4–6 kW (housing flats in Singapore, where 80% of the people lives in), a cost effective home ESS can be achieved by installing a 2 kW Home-ESS in our design with 4 h of backup or 8 kWh. It is important to select a safe battery design that does not create fire risk to the homes. The following design shows how a typical Home-ESS can be wired and operated in two modes, i.e., *Grid Only* and Via *Home ESS* [5]. This design allows the UPS to be bypassed using the static switch for maintenance as shown below in Figure 4. 

The HESS comes with a built-in battery management system that can monitor and control the charging/discharging via the HEMS and the upstream EMS SCADA. It also records the number of cycles that the battery is activated, battery state of charge, charging/discharging status of battery, operating mode of battery, etc. All these parameters allow us to estimate the remaining battery lifetime and its continued utility in supporting grid stabilization efforts as with any large ESS. 

To compare costs, we set up 1200 homes of 2 kW, 8 kWh HESS to make an equivalent High Energy type ESS of 4.8 MW, 9.6 MWh when combined, costing around 8 MUSD in total or around USD5000 each. This is similar in equipment costs except that there is little setup cost, no space needs for the utility and no other related costs such as cabling upgrades. By combining the IL loads with our HEMS, it can be used for high-speed frequency stabilization or demand management, similar to a High Power type ESS at much lower costs with no issue in getting parts for replacements. Simply, it is more cost effective to setup HEMS with IL, with many distributed storage areas in each home to achieve the capacity needed for frequency stabilization or demand management in a simple way that no single or large number of ESS can provide [20,21]. 

## 4. HEMS Modeling

In this section, we describe the design requirements of our HEMS. HEMS comprises smart DB and smart sockets and also includes NILM framework. Safety concerns and minimum changes in existing wiring infrastructure are our primary design concerns. Moreover, it is also critical that the smart DB, smart sockets and NILM are collectively able to identify loads that can participate in the required high speed load shedding needed to balance the grid in safe way. 

### 4.1. Smart DB 

**Figure 5:**
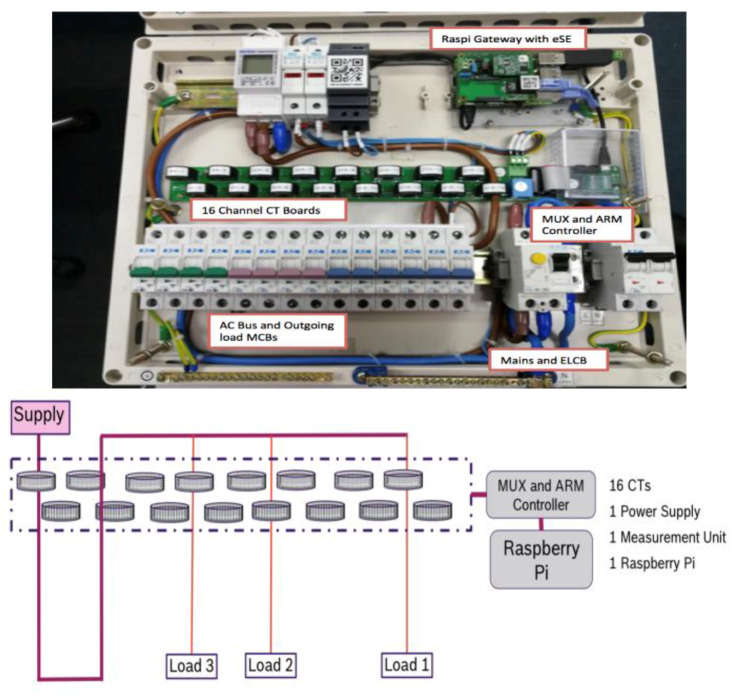
Smart DB layout and Schematic of 16 CT sensor boards.

Smart DB consists of 16 high-speed individual metering CTs for the incoming and each of the out-going circuits to record the voltage current, energy, power and frequency of all circuits. Most importantly, it has a high speed waveform sampling module to record at 6 Khz (6000 samples per second) active and reactive power values to form a load signature be used to identify each home appliance, their on–off timings and with the signature, breakdown of energy usage in terms of each appliance. The layout of the smart DB, NILM MUX controller and the HEMS Raspi Gateway are shown below with a schematic of the layout of the CT sensing board and the schematic diagram of the Current Transformer (CT) board in shown below in Figure 5 below

**Figure 6:**
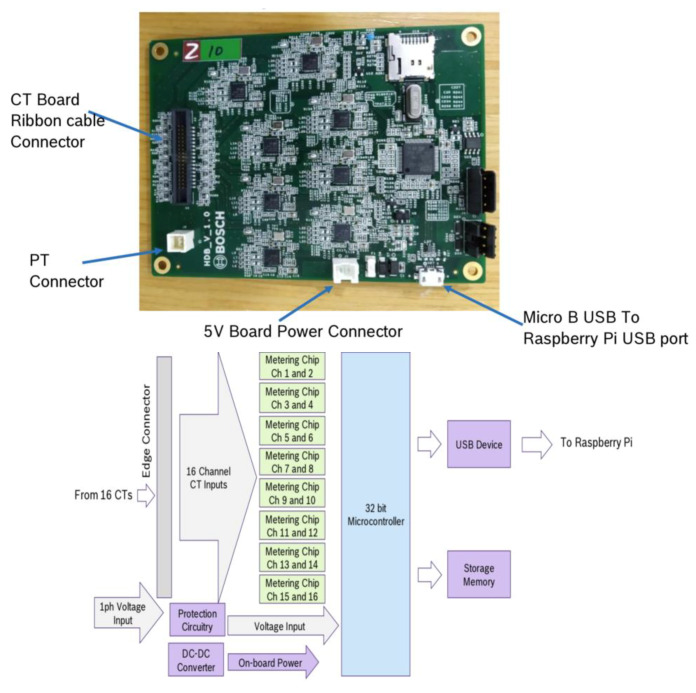
MUX and ARM Controller and the dataflow diagram from the CT to the ARM Controller and Raspi.

From the actual DB layout and the schematic, the CT are spaced to avoid interferences and examples of load 1, 2 and 3 where different circuits from the main AC bus are wired through each CT to measure the loads. With the high speed CT sampling, we can then provide the waveform to the MUX and ARM controllers to handle the waveform processing. This is where the NILM load desegregation application resides to identify the appliance and load usage before sending it to the Raspi gateway. The following Figure 6 shows the components of the MUX and ARM controllers.

This is the controller card that interfaces with the CT sensing boards and the Raspi gateway. Every CT channel correlates with an individual metering chip for individual sensing and waveform capturing.

### 4.2. NILM

Using the information captured by smart DB with the help of various CTs, the objective of NILM algorithms is to identify the type of appliance. We use signature matching technique for appliance identification. In this technique, the captured profile (current, voltage, active power, reactive power, etc.) is matched with already stored profiles in a database and then the closest match is found. This database of signatures can also be gradually built, if not already available. 

As new appliances are identified their signatures are added to the database. The user is also made aware about energy consumption of various appliances thus allowing them to make demand management choices [22]. The following are the test scenarios for learning and identification process of the NILM program in our laboratory. The loads tested included typical home appliances such as a toaster, coffee machines, kettle, vacuum cleaners etc. (loads higher than 150 W).

**Figure 7:**
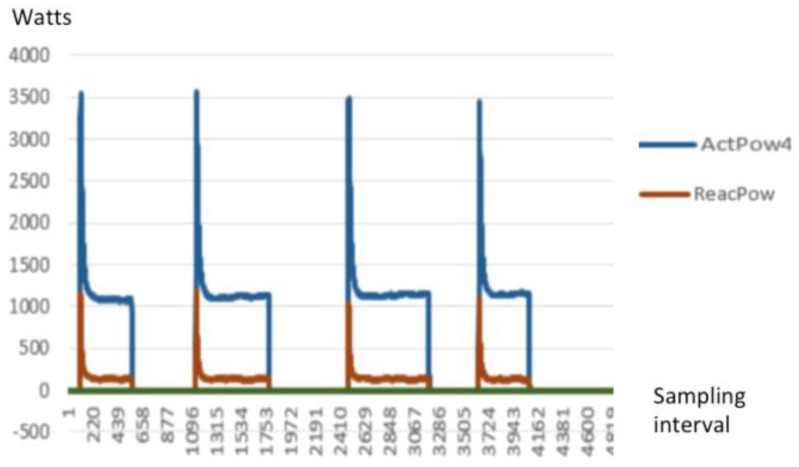
Power (W) signature of a vacuum cleaner.

The following Figure 7 is a typical signature of a high-speed waveform capture of a vacuum cleaner.

The high-speed waveform captured of the active power (blue line) and reactive power (orange line) against the time based sampling interval. As the sampling rate is 6000 samples, the graph is plotted against the sampling interval number captured. For example: at the 4600 sampling interval, the actual time is 4600 × 1/6000 = 0.76 s.

**Figure 8:**
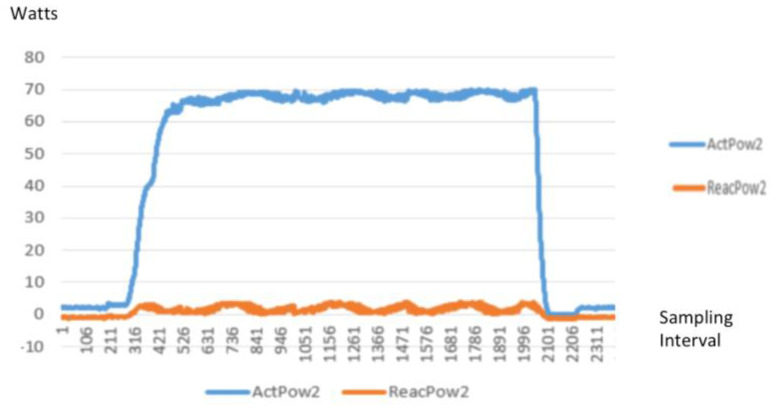
Power (W) signature of an air purifier.

**Figure 9:**
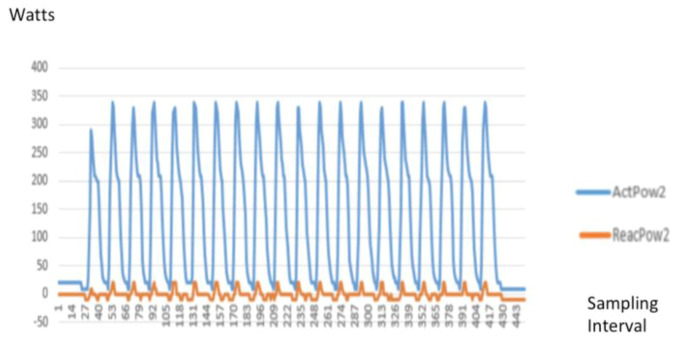
Power (W) signature of a washing machine.

To demonstrate the difference in such waveforms captured, the following Figure 8 and Figure 9 are different profiles or signatures of different household loads.

In order to create a library of signatures, we need to understand that the NILM data collected is based on a time series data for any classification purpose.

$$\begin{array}{c} {\tilde{x}}_{t}=\sum_{n}^{N}\tilde{x}n_{1}t\\ {\tilde{x}}_{t}=\sum_{n=1}^{N}\sum_{k=1}^{K_{m}}Z_{t_{1}}^{n}k\mu_{k}^{n} \end{array}$$

where *N* is the number of types of equipment, *Xn,t* is the power of equipment *n* at time *t*, *X_t_* is the aggregated signal at time *t*. *K_n_* is the appliance different state, *μ_k_* is the power consumed and *Z_t,k_* ∈ {0, 1}. 

**Figure 10:**
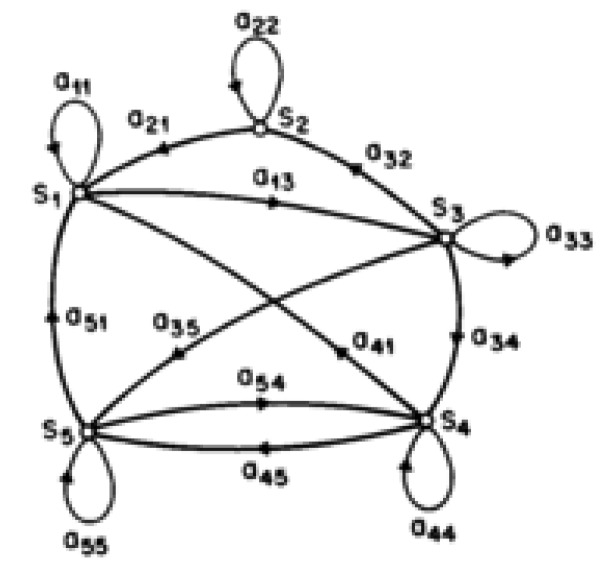
A Markov chain example

The formula in [23] forms the basis for preparing the input file for its signature profile of each appliance. Previously, [24] adopted an approach where every household appliance’s change in power represents a step change in its power values and every appliance stage is a markov state. Using an iterative Hidden Markov Model (HMM), it attempts to model the household’s specific appliances by using its known state appliance’s state and its power demand to create an identity matric. Consider the following Markov Chain Example shown in Figure 10: 

The Markov chain shown indicates that the output is observable, but its states are hidden and just a probabilistic function of the output, making the model a non-observable process normally used for time series processing. However, there are limitations, as complexity increases when there are large numbers of appliances and it does not consider possible simultaneous transitions from different individual appliances, thus limiting its adoption.

In order to address such issue, Factorial Hidden Markov Models (FHMM) based on the first order Markov process was adopted first by [25], that simply considers every observed output such as an additive function of many hidden states. Simply, this model treats independent HMM that are produced in parallel for each monitored device. The aggregated data seen as a whole are taken as the observable output. This is done by combining all the HMM models generated by the appliance in the hidden states and the appliance identification algorithm models which sequence of Markov hidden states could have produced that output to identify it [26,27]. It also suffers from

- Time complexity when the number of appliances, hidden states and the length of observation sequence need to be extended that reduces the classification performance of the model.
- Difficulty in classifying the multistate appliance requiring a long-range pattern resulting the existing models that are based on the first-order Markov process could not be trained efficiently.
- Error in classifying the appliances having similar power consumption.

In this paper, we just adopt the FHMM method and a high-speed 6 kHz sampling rate CT and a separate MUX and Arm controller to address the known issues. 

First, the increased computing power provided by the separate controller allows for it to handle more appliances without affecting its classification performances. Secondly, by being able to capture highly accurate (Class 0.5) accuracy values at 6000 samples in 1 s, it is able to produce a distinct power change signature of each load, avoiding a need for long range pattern, However, it does have limited accuracy when the appliances have similar power consumptions and types of loads such as motor loads that are found in many appliances. 

**Figure 11:**
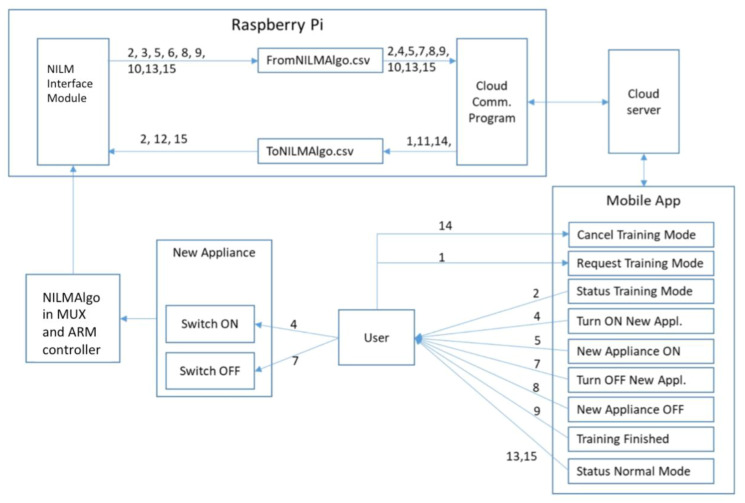
Data Flow of Non-Intrusive Load Monitoring (NILM) Program.

The system can trigger a learning mode where the appliance is explicitly trained by getting the end user to identify the load for the first time by triggering the training mode using the phone app to the controller. Once the training mode is on, the user will be asked to turn on and off the load at least 3 times to capture multiple signatures of the same appliance for identification purposes, based on the FHMM. During the normal operations, the signature will be used to compare against the absolute output previously recorded. This method of direct comparison is accurate within a tolerance of 5% of error is easy, fast and makes the adoption simpler for each household at the start. However, it does not cater for long term appliance pattern change due to appliance degradation, which some adopted using recurrent neural network as part of a deep learning process adopted to address this issue [28]. As of now, the system can trigger the learning mode to update the outputs and adjust to the widening error if needed. More research is needed to know the frequency and period needed for re-training. The data flow diagram is shown in Figure 11.

There is a self-learning program that will learn any new appliance or signature that was previously not known. At each of the number shown in Figure 11 are the set points used by the NILM algorithm to form the profile of each appliance uses changes in active and reactive power. Repeated profiles of the same appliance for up to 3 times within 30 days will trigger to be presented to the user to ask for the specific appliance name. 

In both processes, the identification process is always run first and checked against a known database stored in the local raspi and then the cloud, which is regularly updated. If it is unknown, it goes back to the learning process as a new load. With the appliance identified and their on/off status, the program will add the usage from the moment the appliance starts and stop to the same device appliance. Over time, the accumulated and interval energy usage of each appliance is computed.

The assumption we have is that the load signature is generally constant and continuously triggering the training mode allows it to update the program to update as the devices aged. 

Test Scenarios:

1. Learning and Detection of Toaster (800 watt), Kettle (2300 watt), and Vacuum Cleaner (1200 watt).
2. (Sequence 1) Detection of Toaster, Kettle, and Vacuum Cleaner during Subsequent ON/OFF.
3. (Sequence 2) Detection of Toaster, Kettle, and Vacuum Cleaner during Subsequent ON/OFF.

**Figure 12:**
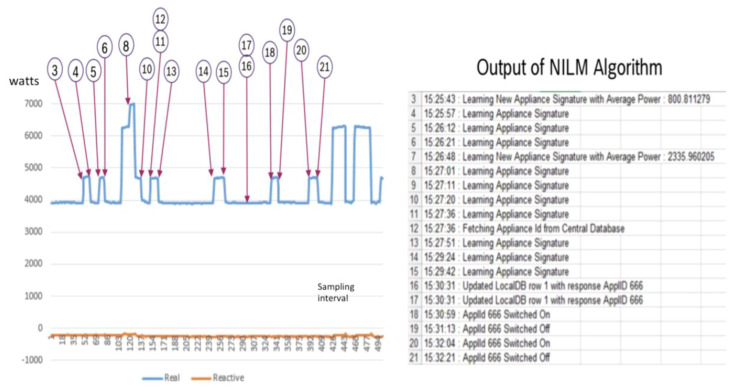
NILM learns new signatures of load appliances.

In Figure 12, the NILM is in training mode and attempts to learn a new appliance signature as shown in item 3 through 11, while at item 12, it fetches a new Appliance ID from the cloud central database for the load learned. At item 7, it learns after at least 3 previous signatures to learn a New Appliance and at item 16, it updates the local DB and at item 18 to 21, it is able to identify the new Appliance ID 666 being switch on and off. The following table shows the simulations of appliances against pretrained modules of NILM of each load and their error levels. 

Many types of typical home appliances are tested and generally, appliances with heater or resistive types of loads are more accurate as compared to those that uses power electronics or switch mode power supply. For loads that are less than 150 W, there are usually difficult to identify as it does not produce significant signature against the background noises generated by all the appliances in operations. The testing was done by turning one appliance on/off at each time.

For loads exceeding 150 W, the accuracy of 1000 tests are known to be almost 95%. It must be qualified that the aircon and water heater are not included because a dedicated circuit was designed to handle it. With NILM, users can understand their usage behavior and set their preferences for load tripping. For example, a certain user may record the following priority for the shedding of loads in his home.

1. Air conditioner—Priority 1
2. Water Heater—Priority 2
3. Kettle—Priority 3
4. Fan—Priority 4
5. Lights—Priority 5

Generally, incentives are higher for higher priority loads. For example, by setting the air con at priority 1, the discounts are higher for all consumption by the air con and the breakdown can be measured by the dedicated circuit and verified by the NILM. This makes it attractive for voluntary participation of interruptible loads (ILs) in our proposed scheme. However, at the same time, utility retain the right to shed specific types of loads, if policy allows, and more than the preferred load list of a user to avoid resorting to the under-frequency load shedding affecting whole regions. Nonetheless, by knowing what is critical and what is not as deemed by the consumers with the help of NILM, the depth of load shedding can be done with minimum inconveniences and with wider choices [20,21]. 

**Figure 13:**
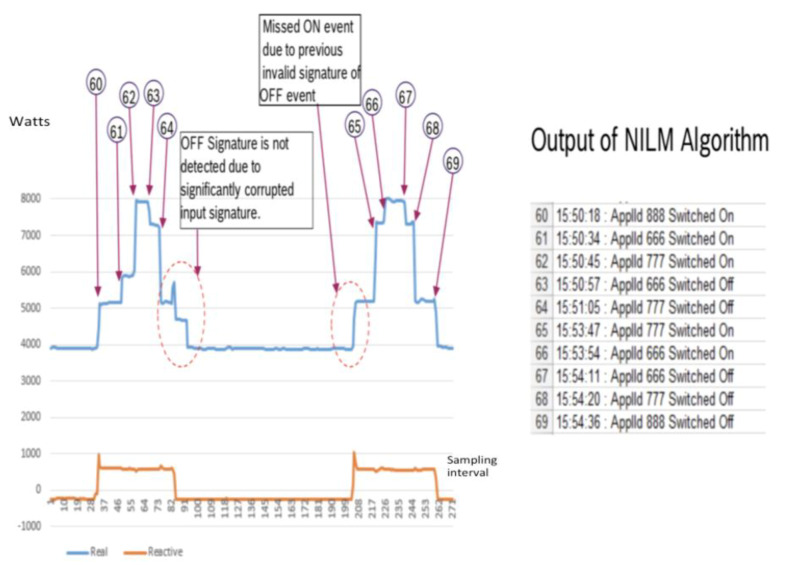
The load identification of different loads learned by the NILM program.

In Figure 13, it shows the high speed identification offered by the approach where all the different appliances are already pretrained and from item 60 to 69, it is able to identify the operations of the appliances being on and off. This is critical as any IL will only make sense to the system if it is being turned on during the load shed operations. Note that the identification is done using the power at the x-axis and the sampling interval where every interval is 1/6000 s. 

Mass quantities of appliances can aggregate using this approach by summing all the appliances under different priorities controlled by the EMS SCADA by issuing load shed commands to the HEMS. A successful Interruptible Load Management System (ILMS) can only be possible if it allows easy participation and when activated, minimize any inconvenience to the end users.

### 4.3. Smart Sockets (SSO)

**Figure 14:**
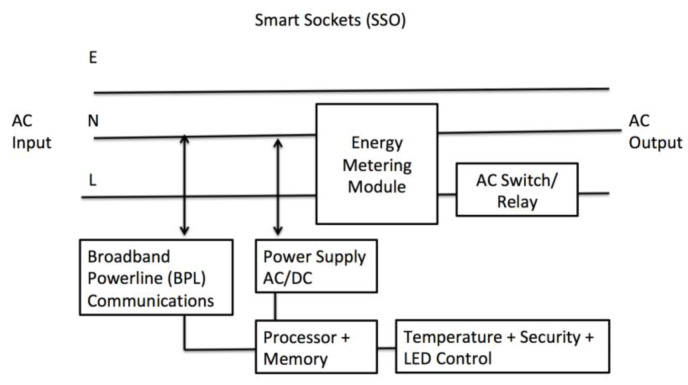
Smart Sockets with built-in temperature sensors. Relay and broadband powerline (BPL) communications.

Smart sockets are used to provide breaker control to individual loads when the demand management decision is made. With this smart SSO, it makes it possible to curtail specific appliances. By using BPL, the breaking of all socket loads can be done within 1–2 s. A typical smart socket with built-in relay and BPL is shown below in Figure 14.

With the help of a smartphone app, the user can specify their load priorities and tripping intervals to allow the EMS SCADA to compute the shedding capacity across the grid at different times. However, actual monitoring through NILM is important to ensure that the load indicated by the user is actually available in his home and operating behind a smart socket at that interval to allow it to actually participate. Therefore, accurate estimation of how much load is offered by the user and how much is actually online when grid stabilization is required are important to know the actual load shedding amount. 

The appliance load shedding commands from EMS SCADA to HEMS can be communicated using internet or loaded into the smart DB against the system frequency [9,12] for under-frequency tripping. These modes are discussed in the next section. 

## 5. HEMS Aggregation Modes for Grid Stabilization 

We propose a two-pronged control approach for energy management.

### 5.1. Centralized Approach

EMS SCADA lists and aggregates all the Home-ESS capacity depending on the current state of charge of each Home-ESS. It also aggregates the load of all the appliances, which are indicated by participating HEMS for load shedding according to the appliance priorities and the indicated duration. This aggregation allows the EMS SCADA to know the total available load shedding capacity. Further information about local weather conditions, total load demand, SCADA generation data are also collected. With this information, ESS SCADA can easily know the difference between the generation capacity and forecasted load demand and the requirement of additional support from aggregated Home-ESS. To minimize user inconvenience, EMS SCADA first discharges enough Home-ESS to achieve stability. If further support is required, it would then start tripping appliances according to the indicated priority list. 

### 5.2. Autonomous Approach

Autonomous approach further simplifies the grid stabilization process. This novel approach, when there is loss of communications, operates based on the decay detection of local supply frequency and user settings of the load priorities for demand management simplifies the management of large number of distributed ESS. In this mode, the frequency threshold values and the action list is published by the EMS SCADA and is broadcast to all the HEMS. Once the HEMS have obtained this list, it uses the home network as the primary network to allow the EMS SCADA to control load curtailment at each home. This autonomous approach allows certain robustness in case of communication loss or for simplicity in management by having a self-autonomous mode of operation. The settings provided by EMS SCADA are locally adjusted by each HEMS according to the state of charge of its Home-ESS and its own appliance priorities. 

An example setting list could be the following:

- 49.5 Hz—Discharge of the Home-ESS
- 49.4 Hz—Tripping of Priority 1 loads
- 49.3 Hz—Tripping of the Priority 2 loads
- 49.2 Hz—until 48.4 Hz—Tripping of the Priority 10 loads
- 48.0 Hz—Substation Relay.

Note that priority level 1 means the first load to be shed and many levels can be set. 

Our two-pronged planning and control (centralized or autonomous modes) strategy allows the utility a novel way to avoid substation level load shedding that indiscriminately affects every households and offers a better way to manage demand growth, improve network stability and offer a better way to affect user usage behavior. Currently in Singapore, the Interruptible Load Scheme available is only offered to industrial customers with each interruptible load coming with a min of 100 kW. Their incentives are peg to the prices offered for the spinning reserve markets based on primary reserve (8 s to trip), secondary reserves (10 min to trip) and contingency reserves (30 min to trip). The reasonably fast response time of our HEMS makes our approach suitable for the more demanding primary, secondary spinning reserve markets, and for those with high-speed communications or utilizing our Smart DB under-frequency tripping, as part of the overall load shedding scheme for frequency stabilization, which currently is not priced as this strategy has never been adopted. This potentially create a more vibrant interruptible load schemes by allowing end consumers to participate as interruptible loads, by aggregating many different home loads with their preferences, as part of the spinning reserve markets [22].

The inclusion of Home-ESS in our proposed scheme also provides a huge capacity already readily available for demand management with several added benefits, e.g., absorbing excess and buffer solar intermittency and allowing a more widespread solar adoption with minimal impact to the grid. The demand response potential of our scheme is also huge. This is shown in our earlier survey for 500 homes that consumers are willing to participate as long as they can set their preferences of when and what they want to participate, although the capacity at home during the industrial peak timing between 10 am to 2 pm is lower compared to the other hours. A simple calculation can be easily shown by adding 2 kW Home-ESS capacity with possible load interruption of up to 1 kW of loads in each home giving a total capacity of 3 kW of demand management for up to 2 h. With 1 million homes, a distributed ESS capacity of 3000 MW with 6000 MWh for instantaneous dispatch can easily reduce up to 45% of Singapore peak usage of 6600 MW for 2 h or staggered across all users reducing the impact and average duration of appliance disruption to each household. In many countries with insufficient capacity, it can assist in reducing investments in generation capacities leadings to huge economic and environment benefits by delaying any such investments, if required at all. 

### 5.3. Security of This System

**Figure 15:**
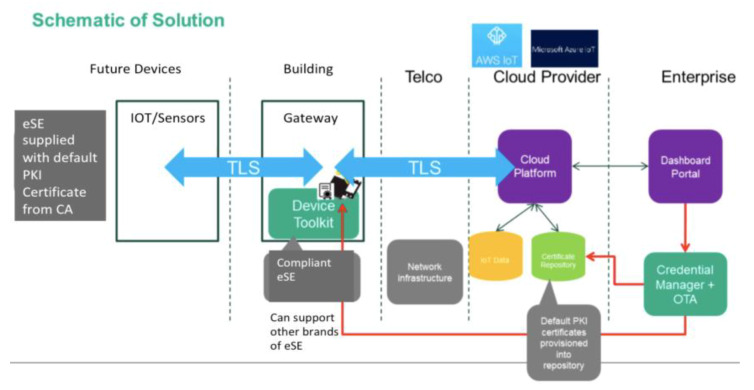
Schematic of the security system.

The system adopted are highly secure and adopts an end to end security design, as shown in Figure 15, by adopting:

- PKI Infrastructure CA and PKI servers based on RSA2048 encryption on the data and Transport Layer Security TLS communication between the gateway and device data collection layer.
- Each gateway consists of a secure element (eSE) to store the signed SSL certificates in each gateway, which is tamperproof
- Use of session keys for every transaction to upload data
- SHA-256 hashing for all information.
- Reviewed by Cybersecurity Agency of Singapore (CSA) and the customer internal security department.
- All sensors also adopt the same TLS architecture

**Figure 16:**
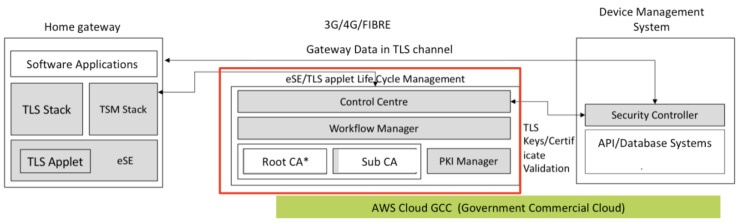
TLS Architecture of the smart DB.

From Figure 16 shown, all communications are secured using TLS communication and all the devices are provisioned via an internal process where the SSL are signed and loaded into the eSE before it is issued to the device gateway to be assembled. All the keys are stored at the HSM provided at the backend. We have provided a security controller to manage all the keys with the PKI server, which is used to manage the expiration and revocation of the keys, when needed. Through this design, we only allow devices that have our signed certificates to connect, encrypt all data that passes between the front gateway and backend based on the session key generated. For ease of management, we set the expiry of the keys with a 10-year limit in time for the hardware renewal.

For user privacy, the signature profile of the appliance is stored locally and in the cloud. We only collect the energy data of each household, subject to their agreement for using the system. No users’ personal information was collected and only the unit address against the data was collected.

## 6. Case Study

A trial is done based on the potential of setting up smart home for 3000+ HDB households with Home-ESS, smart DB, NILM and 10 smart sockets/smart plugs in each home. Please note that there are 1.38 million households in Singapore and 3096 households only represent 0.21% of total households. The purpose of this trial is to demonstrate that even with a tiny participation of households, we can easily aggregate MW capacity and with the two pronged frequency stabilization mechanism, we can easily cut off selective load according to user preferences, provide a huge capacity for demand management and avoid load shedding at the substation levels. 

By considering a typical loads and capacity, the following approach can easily obtain thousands of MW of ESS capacity with a much larger capacity that we can obtain for demand management via our HEMS system. In Table 1 we give a typical system size and the amount of load that can be cut according to user priority, assuming 100% Home-ESS state of charge. 

In the Table 1 and Table 2 above, the EMS SCADA can aggregate 6–10 MW or more power across 3000 homes, which would have required substantial system wiring and land space to achieve similar ESS capacity at the substation level. By using a Smart DB with high speed CTs and NILM, the information makes it possible for households to participate, allow the grid to verify the availability of each appliance and simply varying the incentives for participation to attract more participants. This study demonstrates that the proposed approach provides a novel alternative design approach for utilities to achieve demand management schemes easily as compared to the approach for large scale ESS currently deployed, costing less while making the grid smarter and more user friendly.

For every 1 million households equipped with our system, it would translate to 4000 MW (8000 MWh based on 2 h of discharge), representing around 65–70% of Singapore’s peak power demand of 6600–6800 MW. This would save huge investments in power generation or large scale ESS to balance an ever-growing demand and expectations of a more stable grid, notwithstanding the value add for offering critical services with an UPS installed in the home.

With this usage information on each key load appliances, the system will lead to even more users to make informed choices with their preferences, changing their usage behavior to participate in voluntary demand management participation as our survey suggested. Any methods that can increase user participation either via NILM to understand their own usage, indicate their preferences thus allowing a secondary market aggregation of all interruptible loads for trading of incentives with buildings to avoid paying penalties for exceeding their contract capacities, offers a real solution against any known methods of demand management that are currently deployed. As of now, the Singapore ILMS market has less than 10 participants of facility owners after 14 years of deployment and not open to end home consumers. With our proposed approach, it opens up many possibilities for different Demand Management operators to target small consumers with different possibilities across different geographical locations by aggregating them to meet the minimum requirement of at least 100 kW before they can participate in ILMS offering.

In this approach, a utility managed HEMS have the following cost factors and consideration:

- Cost of setting up the smart DB and gateway hardware in every home.
- Cost in managing and maintaining a utility wide smart home gateway and system that is “after the meter”, which is traditionally not the utility space and cost benefits must be recovered in terms of savings from having to pay for higher spinning reserve capacity to ensure the stability of the network.

Applicability when scaling up:

- The system is highly scalable as utility revenue metering, currently recorded every 30 min or even bi-monthly for mechanical meters can now be broken down into higher resolution or even real time streaming when at home without loading on the utility server as each user will be polling its own gateway.
- Granularity of load usage profile of every appliance will lead to higher customer trusts and satisfaction on their monthly bill as it leads to higher transparency.
- Condition monitoring of appliances when compared to similar usage periods for possible degradation.
- Ability to provide additional services for new revenue stream for the utility.
- Payback model for setting up Home ESS can include other services such as providing a better quality of supply for home critical care services.

Some of the challenges are:

- Need to develop a custom made smart DB to house all the required components and to get the type testing approvals are not easy.
- Use of different communication protocols that must allow the offering of additional sensors and services not part of the utility space.
- Accuracy of the NILM for smaller loads (<100 W) which can be easily mixed up with the background noise.
- Designing a unified security plan for all sub-components.
- Selection of the recording and uploading interval, implementing the schema on the amount of data to upload to avoid using too much bandwidth to the end user. Hence the 6 kHz sampling interval is only locally captured and used to identified the load signature and discarded to avoid collecting too much data. V, I, kW, kVar, kWh and frequency appliances are recorded and uploaded only every 15 min to limit the amount of data. Each file is less than 1 kbyte each and only takes less than 1–2 s to upload on a typical home WIFI of around 100–200 MBits. However, when loads are turn on/off, which affects the ILMS capacity available, these status changes are uploaded immediately. On a grand scale, load changes across thousands of households changes gradually as appliances are being on/off only a few times a day. Even turning on/off the TV load 100 times a day is considered nothing to the system over 24 h.

## 7. Conclusions

In this paper, we proposed a new system design that comprises the use of high-speed sensors such as CT and sockets to make the grid smarter. By aggregating them and integrating them with small capacity Home-ESS coupled with selective load tripping in homes while taking in user priorities, it allows for a more vibrant and intuitive way for grid stabilization that is likely to attract a higher user participation rate for demand response as part of a larger ILMS. We outlined the key design requirements of Home-ESS, HEMS, and the EMS SCADA. The proposed system has the ability to operate in centralized as well as in autonomous modes where it can discharge Home-ESS and shed enough load depending on grid frequency. The fast response time of the proposed scheme makes it a low cost viable alternative in spinning reserve markets. This approach, while providing huge savings in grid investments and achieving huge aggregation of demand capacity at different locations, also makes it more effective than any existing load shedding schemes to balance the grid, while minimizing inconveniences by taking their load priorities into consideration.

## Figures and Tables

**Table 1:** Simulations of NILM against known appliances.

Types of Loads	No. of NILM Simulations	No. Error Cases	% of Error
Above 150W			
Central AC for large hooms	1000	298	29.8%
Room AC/Split Type	1000	321	32.1%
AC Heat Pump	1000	13	1.3%
Kettle/Electric heater	1000	35	3.5%
Dishwasher	1000	328	32.8%
Freezer	1000	16	1.6%
Refrigerator	1000	12	1.2%
Second refrigerator	1000	15	1.5%
Hot water heater	1000	18	1.8%
Tankless Hot Water Heater	1000	30	3.0%
Heat Pump Water heater	1000	3	0.3%
Washing Machine	1000	86	8.6%
Electric Dryer/Hair dryer	1000	68	6.8%
EV Charger	1000	56	5.6%
LED TV	1000	389	38.9%
Toaster	1000	11	1.1%
Microwave Oven	1000	38	3.8%
Below 150 W			
Fan	1000	530	53.0%
Dehumidifier	1000	543	54.3%
Lights	1000	679	67.9%
Aquarium Water Pump	1000	235	23.5%

**Table 2:** Aggregation of HESS and Voluntary Demand Management capacity.

Number of Homes	3000
Available power Home-ESS	2 kW
Available energy Home-ESS	5 kWh
Peak power (3000 homes)	6 MW
Home-ESS State of charge	100%
Priority load 1-Aircon	2 kW
Priority load 2-Water Heater	1 kW
Priority Load 3-Kettle	1.2 kW
Priority load 4-Vacuum Cleaner	0.8 kW
